# Evidence Mapping Based on Systematic Reviews of Repetitive Transcranial Magnetic Stimulation on the Motor Cortex for Neuropathic Pain

**DOI:** 10.3389/fnhum.2021.743846

**Published:** 2022-02-16

**Authors:** Yaning Zang, Yongni Zhang, Xigui Lai, Yujie Yang, Jiabao Guo, Shanshan Gu, Yi Zhu

**Affiliations:** ^1^Department of Kinesiology, Shanghai University of Sport, Shanghai, China; ^2^School of Health Sciences, Duquesne University, Pittsburgh, PA, United States; ^3^Centre for Regenerative Medicine and Health, Hong Kong Institute of Science & Innovation, Chinese Academy of Sciences Limited, Hong Kong, Hong Kong SAR, China; ^4^Department of Rehabilitation Medicine, The Second School of Clinical Medicine, Xuzhou Medical University, Xuzhou, China; ^5^Department of Physical Therapy, University of Toronto, Toronto, ON, Canada; ^6^Department of Musculoskeletal Pain Rehabilitation, The Fifth Affiliated Hospital of Zhengzhou University, Zhengzhou, China

**Keywords:** neuropathic pain, non-pharmacological, repetitive transcranial magnetic stimulation, evidence mapping, evidence synthesis, motor cortex

## Abstract

**Background and Objective:**

There is vast published literature proposing repetitive transcranial magnetic stimulation (rTMS) technology on the motor cortex (M1) for the treatment of neuropathic pain (NP). Systematic reviews (SRs) focus on a specific problem and do not provide a comprehensive overview of a research area. This study aimed to summarize and analyze the evidence of rTMS on the M1 for NP treatment through a new synthesis method called evidence mapping.

**Methods:**

Searches were conducted in PubMed, EMBASE, Epistemonikos, and The Cochrane Library to identify the studies that summarized the effectiveness of rTMS for NP. The study type was restricted to SRs with or without meta-analysis. All literature published before January 23, 2021, was included. Two reviewers independently screened the literature, assessed the methodological quality, and extracted the data. The methodological quality of the included SRs was assessed by using the A Measurement Tool to Assess Systematic Reviews (AMSTAR-2). Data were extracted following a defined population, intervention, comparison, and outcome (PICO) framework from primary studies that included SRs. The same PICO was categorized into PICOs according to interventions [frequency, number of sessions (short: 1–5 sessions, medium: 5–10 sessions, and long: >10 sessions)] and compared. The evidence map was presented in tables and a bubble plot.

**Results:**

A total of 38 SRs met the eligibility criteria. After duplicate primary studies were removed, these reviews included 70 primary studies that met the scope of evidence mapping. According to the AMSTAR-2 assessment, the quality of the included SRs was critically low. Of these studies, 34 SRs scored “critically low” in terms of methodological quality, 2 SR scored “low,” 1 SR scored “moderate,” and 1 SR scored “high.”

**Conclusion:**

Evidence mapping is a useful methodology to provide a comprehensive and reliable overview of studies on rTMS for NP. Evidence mapping also shows that further investigations are necessary to highlight the optimal stimulation protocols and standardize all parameters to fill the evidence gaps of rTMS. Given that the methodological quality of most included SRs was “critically low,” further investigations are advised to improve the methodological quality and the reporting process of SRs.

## Introduction

Neuropathic pain (NP) is an ongoing and challenging condition due to its high morbidity rate of 7–10% in the general population; it usually results from lesions in the somatosensory nervous system, including the peripheral or central nervous system (Colloca et al., 2017). NP negatively impacts patients’ quality of life by reducing functional mobility, activities of daily living, and participation in social roles, which may lead to psychological problems (Kalia and O’Connor, 2005; Gromisch et al., 2020). The initial treatment applied to NP is generally pharmacotherapy, such as use of antidepressants, anticonvulsants, and opioids (Finnerup et al., 2015). However, even with complex treatment regimens, the results of pharmacological approaches remain unsatisfactory, and some may lead to adverse events, such as toxicity, gastrointestinal events, or increased risk of addiction or drug abuse (Papanas and Ziegler, 2016; Urits et al., 2019). Therefore, non-pharmacological interventions, which are considered safe and effective, have been used to treat NP. Repetitive transcranial magnetic stimulation (rTMS) technology is widely accepted at present as a non-pharmacological intervention for treating NP.

The rTMS technique uses a transient high-intensity magnetic field acting on the cerebral cortex to generate induced currents. It alters the action potential of cortical nerve cells, depolarizes neurons in the targeted brain region, and ultimately leads to neuroplastic changes (Paulus et al., 2013). Stimulation target, frequency, and number of sessions are considered critical variables for analgesic efficacy. In terms of stimulation target, the primary motor cortex (M1) is the most commonly used target of stimulation for clinical treatment and has been the most extensively studied. In the 2020 guidelines for rTMS (Lefaucheur et al., 2020), the M1 was recommended as Level A evidence (definitive efficacy) for the treatment of NP. However, clinical promotion is still limited to some extent due to the heterogeneity of treatment protocols, such as frequency and sessions, and effectiveness among various studies. The European Society of Neurology encourages studies to collect and summarize evidence on the factors affecting these techniques (Cruccu et al., 2016). SRs are a common method for synthesizing research evidence. Nonetheless, SRs tend to address more specific research and practice questions and cannot provide a comprehensive overview of rTMS for NP. For example, the following research gaps are unknown: (1) SRs focus on a specific stimulation type or specific pain type (such as pain after spinal cord injury, post-stroke, and diabetic neuropathy), while research on other pain types needs to be developed. (2) The frequency, duration, and other parameters of interventions collected by the SRs varied, and the large amount of evidence with a lack of summarization and classification may lead to clinicians’ confusion. (3) Differences in the quality between individual trials and SRs contributed to the heterogeneity of the evidence. The same primary study may be included in different SRs, which may yield various conclusions due to varying inclusion criteria.

A novel approach to evidence synthesis research called evidence mapping (Grant and Booth, 2009; Haddaway et al., 2016; Miake-Lye et al., 2016) has been developed. Evidence mapping is designed to provide an overview of a research area by including published SRs. Evidence mapping uses published SRs as units of analysis. In the population, intervention, control, and outcome (PICO) framework, evidence mapping extracts and categorizes these data from primary studies, which are included in the SRs. On the basis of the classification criteria, the obtained PICO is integrated into different PICOs, and the contribution of the number of primary studies related to that classification is also calculated to summarize the current interventions (Petersen et al., 2015). The characteristic of the evidence mapping method is to overcome the limitations of primary studies by using the selection of studies, effect size analysis, and bias evaluation of SRs. Considering that the quality of SRs affects the credibility of the evidence, the same primary studies may be included in SRs of different quality. Various conclusions may be drawn due to different inclusion criteria, such as random and double-blind bias. Therefore, evidence mapping uses AMSTAR-2 to evaluate the quality of SRs and the credibility of the results of SRs (Ballesteros et al., 2017; Madera Anaya et al., 2019). Evidence mapping can be translated into two visual products, namely, tables (general information tables and study-specific characteristic tables) and bubble plots (multidimensional composite presentation of classification criteria, quantity, and quality of evidence), which also provide a descriptive narrative summary of the results (Bragge et al., 2011; Haddaway et al., 2016).

Evidence mapping aims to summarize, identify, and analyze the current available evidence in SRs regarding rTMS on M1 for NP. Collecting and integrating data from primary studies on the basis of SRs provide breadth of evidence. Assessing the quality of SRs provides strength of evidence. This information is provided in a user-friendly manner that helps identify research gaps and assist evidence users in the decision-making process.

## Materials and Methods

### Study Design

**FIGURE 1:**
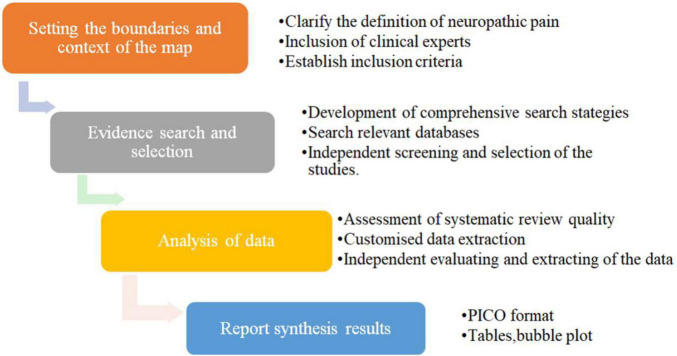
Core tasks performed to map evidence.

Evidence was mapped on the basis of the methodology proposed by Global Evidence Mapping (Bragge et al., 2011). The study process was divided into four phases (Figure 1: Core tasks performed to map evidence).

### Boundaries and Context of Evidence Mapping

Studies and guidelines related to NP were referred, and an expert with research background in NP was consulted to frame the evidence map. With the help of the expert, the specific terminology of the search strategy was confirmed and the possible evidence users (pain, neurology, psychiatry, anesthesiology, and rehabilitation) involved were discussed. On the basis of the above information, the eligibility criteria have been established for inclusion in the study. Studies containing rTMS for NP were considered eligible. Studies on patients with NP were included, whereas experimental subjects that were animals or healthy people were excluded. The intervention should be rTMS, and the comparison could be rTMS, sham rTMS, other treatments of relieving pain, or no treatment. The outcome should be pain measured with various clinically validated tools [e.g., Visual Analog Scale (VAS), Numerical Rating Scale (NRS), Short-Form McGill Pain Questionnaire, and Brief Pain Inventory]. Studies that did not address intervention outcomes, such as those that aimed to explore NP-related pathophysiology and focus on cost-effectiveness, were excluded. Studies that reported other outcomes (e.g., fatigue, motor function, spasticity, sensory function, and cognition) but pain were also excluded. Only SRs (with or without meta-analysis) were included as they provided reliable evidence.

### Evidence Search and Selection

We conducted searches of systematic literature on PubMed, EMBASE, Epistemonikos, and The Cochrane Library Published before January 23, 2021. Medical subject headings (mesh terms), free-text terms, and synonymous terms available for NP and transcranial magnetic stimulation, such as “neuralgia,” “neurodynia,” “atypical neuralgia,” “nerve pain,” and “stump neuralgia,” were combined. Literature published in non-English languages was excluded. References of the relevant studies that met the inclusion criteria were added to potential additional reviews. The details of the search strategies are reported in Supplementary Material 1. EndNote (version X9) was applied to manage the search results. Duplicate SRs were removed, and two reviewers (YZa and XL) independently screened the titles and abstracts to exclude irrelevant studies. Full-text studies were obtained and reviewed to make a terminal decision. Any disagreements in the decision-making process were resolved through negotiation or by discussion with a third reviewer (YoZ).

### Data Analysis

A data extraction table was designed to record the main characteristics and compare the methodological differences of the included SRs. Two authors (YZa and XL) assessed the methodological quality and extracted data independently. Any difference of opinions was discussed with the third author (YoZ). The original authors were contacted for missing information when necessary. Data were grouped into three categories:

(a) The Assessment Methodological Quality for Systematic Reviews (AMSTAR-2) was used to assess the methodological quality of SRs (Shea et al., 2017). AMSTAR-2 is a practical tool used to assess the quality of SRs that include randomized or non-randomized studies of healthcare interventions or both. It has 16 items, with an overall rating based on weaknesses in critical domains (items: 2, 4, 7, 9, 11, 13, and 15). In brief, the evaluation results of the SRs are generally divided into the following four categories: “High,” no critical weakness and no more than one non-critical weakness; “Moderate,” no critical weakness and more than one non-critical weakness; “Low,” one critical flaw with or without non-critical weaknesses; and “Critically low,” more than one critical flaw with or without non-critical weaknesses.

(b) The following characteristics of SRs were extracted: authors, years of publication, types of SR (with or without meta-analysis), objectives, dates of search, sample sizes, designs, and numbers of included studies.

(c) The PICO framework was used to extract data from primary studies included in the SRs. The four key components are populations, intervention, comparison, and outcomes. Details including population characteristics (e.g., NP related to spinal cord injury, post-stroke symptoms, and complex regional pain syndrome), interventions (e.g., target area, frequency, and intensity of transcranial magnetic stimulation), comparative measures (e.g., placebo and sham stimulation), and outcomes were extracted. The obtained data of PICO from primary studies in SRs were classified into different groups based on similar characteristics (population; interventions: frequency, session, and intensity; control group; and outcome scale).

For descriptive purposes, the effect of rTMS on NP reported by the SR authors was grouped into the following five categories on the basis of the previously reported criteria: “Potentially better,” the conclusions reported rTMS as more beneficial than the control group; “Mixed results,” the same primary study had different findings among different studies (e.g., some studies found no difference in rTMS compared with the control group in the same population, whereas others found potential benefits of transcranial magnetic stimulation over the control group); “Unclear,” insufficient evidence to draw definitive conclusions about the effectiveness of rTMS on pain; “No difference,” the conclusions provided evidence of no difference between intervention and control; and “Potentially worse,” the conclusions reported TMS as less beneficial than the control group. The same primary study may be included in multiple SRs. If the primary study has multiple consistent results, it would be added to the appropriate group, and multiple conflicting findings were included in the “Mixed Results” group (Miake-Lye et al., 2019).

### Reporting Synthesized Findings

Each clinical question addressed in each included review was adapted into a PICO format that specified the types of participants, interventions (or comparison), and outcomes. Evidence mapping allows the reader to visualize any gaps in the literature base, with results presented in the form of tables and graphs.

(a) The basic characteristics, quality assessment of the included SRs, and characteristics of all integrated PICOs were described in tables.
(b) A heat map was used to present the quality of the included SRs.
(c) Graphical display was provided through bubble plots. The bubble plot displayed information in four dimensions: (1) bubble size (number of articles), the size of each bubble is proportional to the number of individual trials included in the SRs; (2) bubble color (research characteristics), bubbles labeled with different colors indicate different PICOs; (3) X-axis (effect of TMS on NP), the classification of authors’ conclusions represented on the X-axis (“potentially better,” “mixed results,” “unclear,” “no difference,” and “worse”); and (4) Y-axis (AMSTAR-2 evaluation results), four different colors were used to indicate study quality, with red indicating critically low, orange indicating low, yellow indicating moderate, and green indicating high quality.

## Results

### Selected Studies

**FIGURE 2:**
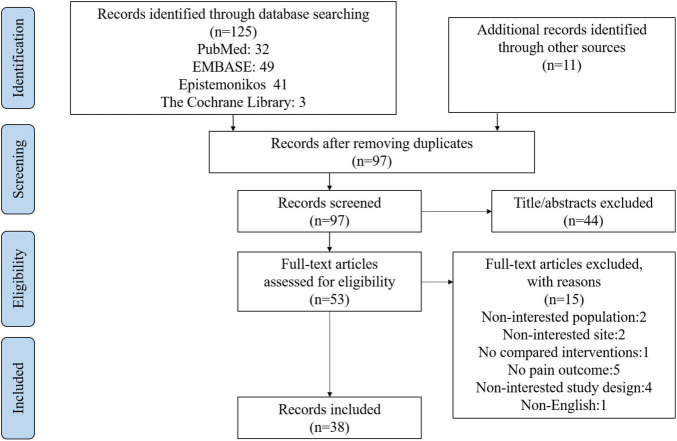
PRISMA flow diagram detailing the selection process.

This retrieval yielded 125 records. Another 11 articles were added from the references that met the inclusion criteria. After duplicates were removed, 97 articles remained for screening of the titles and abstracts. Subsequently, 44 articles were excluded after the screening. In the remaining 53 articles, 13 were excluded after full-text reviews. Finally, 38 articles met the eligibility criteria (Figure 2). The list of excluded studies along with exclusion rationales can be found in Supplementary Material 2.

### Methodological Quality of Systematic Reviews

**FIGURE 3:**
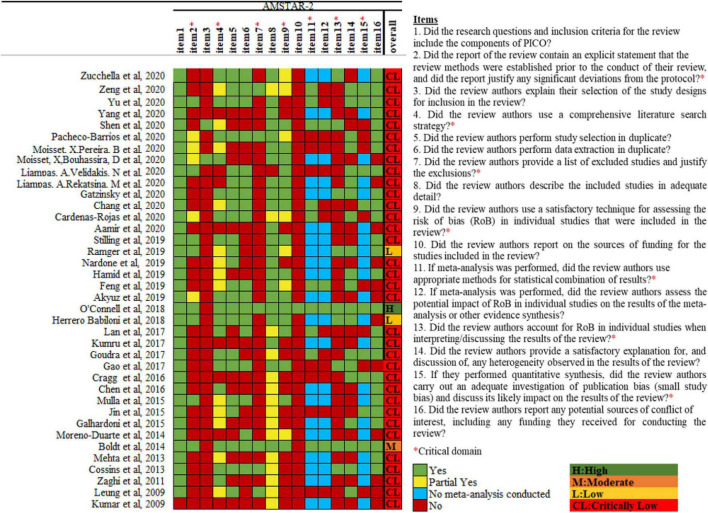
Methodological quality of the included systematic reviews.

According to the AMSTAR-2 criteria, 34 SRs scored “critically low,” 2 SR scored “low,” 1 SR scored “moderate,” and 1 SR scored “high” in terms of methodological quality (Figure 3). The most frequent drawbacks were as follows: no mention of the protocol in the systematic overview, no description of the rationale for the study designs included in the review, no report of excluded studies or reasons for exclusion, and no statement of funding for the included studies. The detailed assessment process is provided in Supplementary Material 3.

### Characteristics of the Included Systematic Reviews

Table 1 shows the characteristics of the included SRs. All SRs were published between 2009 and 2020. Among the 38 included SRs, 17 SRs conducted a meta-analysis. The number of included studies ranged from 3 to 131, and they were conducted between 2001 and 2020. Each SR included patients ranging from 97 to 15,776. Six SRs did not report or incompletely reported the designs of the included individual studies. All studies reported study designs, and a total of 678 randomized controlled trials (RCTs) accounted for 86% of the included studies in all SRs. Of all SRs, 18 SRs included only RCTs, 15 SRs included patients with NP with different underlying causes, and 25 were exclusively conducted on NP with specific etiologies or due to a single disease. Six SRs included pain after spinal cord injury (SCI), another 5 SRs included central post-stroke pain (P), 3 SRs included phantom limb pain (PLP), 2 SRs included migraine, 2 SRs included complex regional pain syndrome (CRPS), 2 SRs included headache, 1 SR included diabetic peripheral neuropathy (DPN), 1 SR included multiple sclerosis (MS), and 1 SR included orofacial pain (OFP). As for the intervention, 13 SRs only assessed rTMS, 12 SRs assessed other non-invasive stimulations, 3 SRs assessed neuromodulation techniques, 4 SRs assessed non-pharmacological interventions, 5 SRs assessed pharmacological and non-pharmacological management of NP, and 1 SR assessed non-invasive brain stimulation combined with exercise.

**TABLE 1:** Characteristics of included systematic reviews.

Author and year	Study design	Search date	Objective	Number of studies included	Design and number of included studies	Participants (n)
Zucchella et al., 2020	SR	February 2020	To evaluate the effect of non-invasive brain and spinal cord stimulation in the treatment of pain in multiple sclerosis	9	RCT: 1 NCS: 6 FP: 1 CR: 1	175
Zeng et al., 2020	SRM	August 2019	To evaluate the effect of NINM in relieving pain intensity and improving nerve conduction velocity in patients with diabetic peripheral neuropathy	20	RCT: 18 QE: 2	1167
Yu et al., 2020	SRM	January 2019	To investigate the effect of non-invasive brain stimulation on NP in patients with SCI	11	RCT: 11	274
Yang and Chang, 2020	SR	June 2019	To explore the effect of rTMS on the control of various types of pain conditions	106	RCT: 69 OLT: 16 CR: 21	3264
Shen et al., 2020	SRM	November 2019	To evaluate the effect of non-invasive brain stimulation (rTMS and tDCS) on NP after SCI	10	RCT: 10	214
Pacheco-Barrios et al., 2020	SRM	February 2019	To assess the efficacy of neuromodulation techniques for the treatment of PLP in adults	14	RCT: 9 QE: 5	261
Moisset et al., 2020a	SRM	July 2020	To investigate the efficacy of neurostimulation techniques in migraine	38	RCT: 38	2899
Moisset et al., 2020b	SR	August 2019	To propose all the alternative treatment options for NP	131	RCT: 131	15776
Liampas et al., 2020a	SRM	November 2019	To describe the prevalence and characteristics of CPSP and investigate the relevant management methods	69	NR	NA
Liampas et al., 2020b	SR	April 2020	To assess the effect of non-pharmacological interventions in the management of peripheral NP	18	RCT: 13 OLT: 5	1613
Gatzinsky et al., 2020	SR	June 2019	To review primary research regarding the efficacy and safety of rTMS on M1	32	RCT: 24 CS: 8	682 (RCT)
Chang et al., 2020	SRM	February 2020	To assess the effectiveness of rTMS in the treatment of CRPS-related pain	3	RCT: 1 Prospective observational studies: 2	41
Cardenas-Rojas et al., 2020	SRM	November 2019	To assess the efficacy and safety of NIBS combined with exercise in the treatment of chronic pain	8	RCT: 8	219
Aamir et al., 2020	SR	June 2019	To evaluate the effect of rTMS in the management of peripheral NP	12	RCT: 5 CS: 2 CR: 5	188
Stilling et al., 2019	SR	September 2018	To review the use of TMS and tDCS for specific headache disorders	34	Randomized trials: 20 NRC/Prospective cohort/OLT: 14	1787
Ramger et al., 2019	SR	2018	To evaluate the efficacy of rTMS and tDCS in the treatment of CPSP	6	RCT: 1 Prospective cohort: 1 CS: 2 Cross-over: 2	109
Nardone et al., 2019	SR	April 2018	To evaluate the efficacy of TMS in treating patients with painful and non-painful phantom phenomena	18	NR	NA
Hamid et al., 2019	SR	2018	To evaluate the effect of rTMS on chronic refractory pain, especially in adults with central NP	12	RCT: 12	350
Feng et al., 2019	SRM	September 2018.	To evaluate the efficacy of rTMS and tDCS in RCTs in the treatment of migraine	9	RCT: 9	276
Akyuz and Giray, 2019	SR	August 2017	To assess the effectiveness and safety of rTMS and tDCS in the treatment of PLP	4	RCT: 4	97
O’Connell et al., 2018	SRM	October 2017	To assess the efficacy of non-invasive cortical stimulation techniques chronic pain	94	RCT: 94	2983
Herrero Babiloni et al., 2018	SR	NR	To evaluate the analgesic effect of TMS and tDCS in the treatment of different etiologies of chronic OFP	14	RCT: 14	228
Lan et al., 2017	SRM	April 2017	To evaluate the efficacy of TMS in the treatment of migraine	5	RCT: 5	313
Kumru et al., 2017	SR	August 2015	To assess the role of rTMS or peripheral magnetic stimulation for the treatment of NP	39	NR	892
Goudra et al., 2017	SRM	NR	To evaluate the effect of rTMS in the management of chronic pain	9	RCT: 6 Prospective observational: 3	183
Gao et al., 2017	SRM	March 2016	To assess the analgesic effect of rTMS in patients with SCI-related NP	6	RCT: 6	127
Cragg et al., 2016	SRM	May 2015	To explore the predictors of placebo responses in central NP clinical trials	39	RCT: 39	1153
Chen et al., 2016	SR	September 2015	To evaluate the antalgic effects of NIPMs on CPSP	16	NA	184
Mulla et al., 2015	SR	December 2013	To provide an overview of the evidence-based management of CPSP	8	RCT: 8	459
Jin et al., 2015	SRM	December 2014	To evaluate the optimal parameters of rTMS for NP	25	RCT: 20 Self-controlled: 5	589
Galhardoni et al., 2015	SR	2014	To review the literature on the analgesic effects of rTMS in chronic pain	33	RCT: 33	842
Moreno-Duarte et al., 2014	SR	2012	To evaluate the effectiveness and safety of neural stimulation techniques for the treatment of SCI pain	10	RCT: 8 OLT: 2	307
Boldt et al., 2014	SRM	March 2011	To review the available research evidence to explore the effectiveness of non-pharmacological interventions in patients with SCI	16	RCT: 16	616
Mehta et al., 2013	SR	April 2011	To review the literature on non-pharmacological treatment of post-SCI pain	17	RCT: 9 Prospective controlled trial: 2 CS: 1 Pre-post: 5	433
Cossins et al., 2013	SR	February 2012	To explore therapeutic methods for effective management of CRPS	29	RCT: 29	NR
Zaghi et al., 2011	SR	January 2010	To review the analgesic efficacy of TMS and tDCS and to discuss potential mechanisms of action	18	NR	413
Leung et al., 2009	SRM	August 2007	To evaluate the overall analgesic effect of rTMS in M1 for NP and evaluate the effect of treatment parameters. such as pulse, frequency, and number of sessions on the treatment effect	5	RCT: 5	149
Kumar et al., 2009	SR	September 2008	To review pathophysiology and treatment of CPSP	NA	NA	NA

*SR, systematic review; SRM, systematic review with meta-analysis; NINM, non-invasive neuromodulation; NP, neuropathic pain; SCI, spinal cord injury; rTMS, repetitive transcranial magnetic stimulation; CPSP, central poststroke pain; tDCS, transcranial direct current stimulation; PLP, phantom limb pain; M1, motor cortex; CRPS, complex regional pain syndrome; NIBS, non-invasive brain stimulation; TMS, transcranial magnetic stimulation; OFP, orofacial pain; NIPMs, non-invasive physical modalities; RCT, randomized controlled trial; NCS, non-controlled trial; FP, feasibility pilot; CR, case report; QE, quasi-experiment; OLT, open-label trial; NR, not reported. NRT, non-randomized trial; CS, case series; NA, not available.*

### Characteristics of Population, Intervention, Comparison, and Outcomes From Systematic Reviews

After merging the duplicate primary studies included in the 38 SRs, 70 primary studies were integrated into 19 PICO groups based on the PICO characteristics. Studies that did not provide the mandatory parameter information were not included in the PICOs. Among the included SRs, populations with NP were from various diseases and etiologies, and the treatment protocols adopted various parameters, including frequency, sessions, and pulses. Sham stimulation or placebo was the most common intervention in the control group of rTMS on the M1. The primary outcome of the included studies was self-reported subjective nociception. VAS and NRS were the most commonly validated pain assessment scales. The details of the characteristics are enumerated in Supplementary Material 4.

**FIGURE 4:**
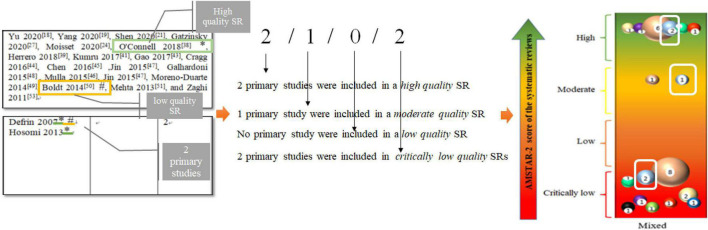
Interpretation of evidence mapping.

As a result of unavoidable heterogeneity of the rTMS protocol among studies, classifying and categorizing all parameters can be difficult. Thus, the classification of PICO focused on interventions and comparison, as well as the population involved and outcome assessments, as presented in Table 2. We use Figure 4 to explain the connection between the bubble polt and Table 2. In terms of interventions, we classified them to frequency and session of rTMS, as they have been shown to influence the analgesic effects and are identified as clinically significant factors (Ahmed et al., 2012; O’Connell et al., 2018; Gatzinsky et al., 2020; Pacheco-Barrios et al., 2020). High and low frequency of rTMS can induce transient excitatory and inhibitory effects, respectively (Klein et al., 2015). Sessions of rTMS are considered an important factor in maintaining the effects. The characteristics of the interventions were categorized based on frequency (low or high frequency) and number of sessions (short: 1–5 sessions, medium: 5–10 sessions, and long: >10 sessions).

**TABLE 2:** PICOs included in systematic reviews.

PICOS number	PICOs in bubble chart	Intervention	Parameters		Comparison	Population	Outcomes	Included SRs	Primary studies included in SRs				Conclusion
				
			Frequency (Hz)	Session schedule					RCT	Observation	Number of primary studies	Number of SRs involving the Quality (high/moderate/low/critically low) of primary studies	
1	50 Hz, short sessions versus sham rTMS	rTMS	50 Hz	1 session	Sham rTMS	Chronic OFP	VAS	Herrero Babiloni et al., 2018	Kohútová et al., 2017		1	0/0/0/1	Potentially better
		rTMS	50 Hz	1 session	Sham rTMS	Chronic OFP	VAS	Herrero Babiloni et al., 2018	Kohútová et al., 2017		1	0/0/0/1	No difference (long term)
2	20 Hz, long sessions versus sham rTMS	rTMS	20 Hz	11 sessions	Sham rTMS	Mixed NP	VAS	Yang and Chang, 2020		Pommier et al., 2016	1	0/0/0/1	Potentially better
3	20 Hz, medium sessions versus sham rTMS	rTMS	20 Hz	10 sessions	Sham rTMS	Pelvic pain (urinary bladder pain syndrome)/cancer and malignant NP	VAS	Gatzinsky et al., 2020; Moisset et al., 2020b; Yang and Chang, 2020	Khedr et al., 2015; Cervigni et al., 2018		2	0/0/0/2	Potentially better
		rTMS	20 Hz	10 sessions	Sham rTMS	Cancer and malignant NP	VAS	Jin et al., 2015; Gatzinsky et al., 2020; Moisset et al., 2020b; Yang and Chang, 2020	Jin et al., 2015; Khedr et al., 2015		2	0/0/0/2	No difference (1 month later)
4	20 Hz, short sessions versus sham rTMSr	TMS	20 Hz	5 sessions	Sham rTMS	LBP, non-specified OFP, TN after dental or neural surgery, atypical facial pain, CPSP, TN, PLP, Alzheimer, TGNP, and IBS	VAS	Kumar et al., 2009; Leung et al., 2009; Zaghi et al., 2011; Galhardoni et al., 2015; Jin et al., 2015; Chen et al., 2016; Goudra et al., 2017; Kumru et al., 2017; Herrero Babiloni et al., 2018; O’Connell et al., 2018*; Akyuz and Giray, 2019; Hamid et al., 2019; Nardone et al., 2019; Aamir et al., 2020; Gatzinsky et al., 2020; Liampas et al., 2020a; Moisset et al., 2020b; Pacheco-Barrios et al., 2020^]^; Yang and Chang, 2020	Khedr et al., 2005*; Ahmed et al., 2011*, 2012; Fricova et al., 2013; Melchior et al., 2014; Ambriz-Tututi et al., 2016		11	4/0/0/11	Potentially better
		rTMS	20 Hz	4 sessions	Sham rTMS	CPSP, SCI	NRS	Yang and Chang, 2020		Quesada et al., 2018			Potentially better
		rTMS	20 Hz	2 sessions	Sham rTMS	Mixed NP	VAS	Galhardoni et al., 2015; Kumru et al., 2017; O’Connell et al., 2018*; Gatzinsky et al., 2020; Yang and Chang, 2020	Andre-Obadia et al., 2008 *				Potentially better (posteroanterior orientation of the coil)
		rTMS	20 Hz	2 sessions	Sham rTMS	Mixed NP and TN	NRS	Herrero Babiloni et al., 2018; Hamid et al., 2019; Yang and Chang, 2020	Andre-Obadia et al., 2018				Potentially better (with M1 hand)
		rTMS	20 Hz	1 session	Sham rTMS	Chronic neuropathic pain (mixed), CPSP, SCI, BPL, and TGNP	NRS	Jin et al., 2015; Kumru et al., 2017; O’Connell et al., 2018*; Hamid et al., 2019; Gatzinsky et al., 2020	Andre-Obadia et al., 2011*, 2014				Potentially better
		rTMS	20 Hz	5 sessions	Sham rTMS	DPN	VAS	Galhardoni et al., 2015; Jin et al., 2015; Kumru et al., 2017; O’Connell et al., 2018*; Aamir et al., 2020; Liampas et al., 2020b; Moisset et al., 2020b; Yang and Chang, 2020; Zeng et al., 2020	Onesti et al., 2013 *		2	1/0/0/2	Mixed
		rTMS	20 Hz	1 session	Sham rTMS	LBP	Pain intensity	Zaghi et al., 2011; Galhardoni et al., 2015	Johnson et al., 2006				Mixed
		rTMS	20 Hz	5 sessions	Sham rTMS	Rectal sensitivity in IBS		Goudra et al., 2017	Melchior et al., 2013		5	3/0/0/5	No difference
		rTMS	20 Hz	3 sessions	Sham rTMS	Mixed NP	VAS	Zaghi et al., 2011; Galhardoni et al., 2015; Jin et al., 2015; O’Connell et al., 2018*; Gatzinsky et al., 2020; Yang and Chang, 2020	Andre-Obadia et al., 2006 *				No difference
		rTMS	20 Hz	2 sessions	Sham rTMS	Mixed NP	VAS	Galhardoni et al., 2015; Jin et al., 2015; Kumru et al., 2017; O’Connell et al., 2018*	Andre-Obadia et al., 2008 *				No difference (lateromedial orientation of the coil)
		rTMS	20 Hz	2 sessions	Sham rTMS	Mixed NP	NRS	Herrero Babiloni et al., 2018	Andre-Obadia et al., 2018				No difference (with M1 face)
		rTMS	20 Hz	1 session	Sham rTMS	Mixed NP	VAS	Leung et al., 2009; Zaghi et al., 2011; Jin et al., 2015; Galhardoni et al., 2015; O’Connell et al., 2018*; Yang and Chang, 2020	Rollnik et al., 2002 *				No difference
5	10 Hz, long sessions versus sham rTMS	rTMS	10 Hz	15 sessions	Sham rTMS	PHN	VAS	Moisset et al., 2020b	Pei et al., 2019		2	0/0/0/2	Potentially better
		rTMS	10 Hz	12 sessions	Sham rTMS	Migraine		Lan et al., 2017	Shehata et al., 2016				Potentially better
		rTMS	10 Hz	12 sessions	Sham rTMS	Migraine	VAS	Stilling et al., 2019; Yang and Chang, 2020	Shehata et al., 2016		1	0/0/0/1	Mixed
6	10 Hz, medium sessions versus sham rTMS	rTMS	10 Hz	10 sessions	Sham rTMS	SCI, chronic central pain after mild traumatic brain injury, thalamic pain, hemiplegic shoulder pain, PHN, PLP, and CPSP	NRS, VAS	Cragg et al., 2016; Gao et al., 2017; O’Connell et al., 2018*; Akyuz and Giray, 2019; Nardone et al., 2019; Aamir et al., 2020; Gatzinsky et al., 2020; Moisset et al., 2020b; Shen et al., 2020; Yang and Chang, 2020	Yilmaz et al., 2014*; Ma et al., 2015; Malavera et al., 2016; Choi et al., 2018; Choi and Chang, 2018	Lin et al., 2018	7	1/0/0/7	Potentially better
		rTMS	10 Hz	9 sessions	Sham rTMS	Mixed NP	VAS	Yang and Chang, 2020	Lawson et al., 2018				Potentially better
		rTMS	10 Hz	10 sessions	Sham rTMS	CRPS	VAS	Cossins et al., 2013; Galhardoni et al., 2015; Jin et al., 2015; O’Connell et al., 2018*; Cardenas-Rojas et al., 2020; Chang et al., 2020; Gatzinsky et al., 2020; Yang and Chang, 2020	Picarelli et al., 2010 *		1	1/0/0/1	Mixed
		rTMS	10 Hz	10 sessions	Sham rTMS	SCI	VAS	Gao et al., 2017; Gatzinsky et al., 2020; Shen et al., 2020; Yang and Chang, 2020	Yilmaz et al., 2014		3	1/0/0/2	No difference (<6 weeks)
		rTMS	10 Hz	10 sessions	Sham rTMS	PLP	VAS	O’Connell et al., 2018*; Aamir et al., 2020; Gatzinsky et al., 2020; Moisset et al., 2020b; Pacheco-Barrios et al., 2020; Yang and Chang, 2020	Malavera et al., 2013**, 2016				No difference (30 days)
		rTMS	10 Hz	10 sessions	Sham rTMS	Migraine	Total HA days; overall HA index	Stilling et al., 2019	Teo et al., 2014		1	0/0/0/1	Potentially worse
7	10 Hz, short sessions versus sham rTMS	rTMS	10 Hz	5 sessions	Sham rTMS	Mixed refractory neuropathic pain, radiculopathy, CPSP, mixed NP, pelvic pain, and facial NP	VAS, NRS	Kumru et al., 2017; O’Connell et al., 2018*; Gatzinsky et al., 2020; Yang and Chang, 2020	Lang et al., 2014; Nurmikko et al., 2016*	Pinot-Monange et al., 2019	18	5/0/0/18	Potentially better
		rTMS	10 Hz	3 sessions	Sham rTMS	Mixed NP, MBTI-HA, chronic migraine, CPSP, SCI, NTL, BPL, PLP, PNI, TGNP, and migraine	VAS, NRS	Zaghi et al., 2011; Galhardoni et al., 2015; Jin et al., 2015; Kumru et al., 2017; Gao et al., 2017; O’Connell et al., 2018*; Feng et al., 2019; Stilling et al., 2019; Gatzinsky et al., 2020; Yang and Chang, 2020	Lefaucheur et al., 2006*, 2008*; Saitoh et al., 2007*; Misra et al., 2013, 2017; Kalita et al., 2016; Leung et al., 2016	Lefaucheur et al., 2006, 2012; Misra et al., 2013; Kalita et al., 2017			Potentially better
		rTMS	10 Hz	1 session	Sham rTMS	Migraine, CPSP, SCI, PNP, mixed NP, CPSP, SCI, trigeminal nerve lesion (failure of TN surgery), BPL, and TGNI	VAS	Kumar et al., 2009; Leung et al., 2009; Zaghi et al., 2011; Galhardoni et al., 2015; Chen et al., 2016; Gao et al., 2017; Kumru et al., 2017; Herrero Babiloni et al., 2018; O’Connell et al., 2018*; Gatzinsky et al., 2020; Moisset et al., 2020a; Yang and Chang, 2020	Lefaucheur et al., 2004*, 2011; Misra et al., 2013, 2017				Potentially better
		rTMS	10 Hz	5 sessions	Sham rTMS	SCI	NRS	Zaghi et al., 2011; Mehta et al., 2013; Boldt et al., 2014^#^; Moreno-Duarte et al., 2014; Galhardoni et al., 2015; Jin et al., 2015; Cragg et al., 2016; Gao et al., 2017; Goudra et al., 2017; Kumru et al., 2017; O’Connell et al., 2018*; Gatzinsky et al., 2020; Moisset et al., 2020b; Shen et al., 2020; Yang and Chang, 2020; Yu et al., 2020	Kang et al., 2009 * ^#^		8	6/1/0/8	Mixed
		rTMS	10 Hz	3 sessions	Sham rTMS	Mixed NP, CPSP, and BPL	VAS	Jin et al., 2015; Gao et al., 2017; Kumru et al., 2017; O’Connell et al., 2018*; Yang and Chang, 2020	Lefaucheur et al., 2001b *	Lefaucheur, 2003			Mixed
		rTMS	10 Hz	2 sessions	Sham rTMS	Mixed NP, radiculopathy, TN after surgery, and atypical facial pain after dental surgery	VAS	Herrero Babiloni et al., 2018; Yang and Chang, 2020	Ayache et al., 2016				Mixed
		rTMS	10 Hz	1 session	Sham rTMS	SCI, CRPS, mixed NP, CPSP, and SCI	VAS, NRS	Cossins et al., 2013; Galhardoni et al., 2015; Jin et al., 2015; Chen et al., 2016^[^; Cragg et al., 2016; Gao et al., 2017; Kumru et al., 2017; Herrero Babiloni et al., 2018; O’Connell et al., 2018*; Chang et al., 2020; Gatzinsky et al., 2020; Shen et al., 2020; Yang and Chang, 2020	Lefaucheur et al., 2001a*; Pleger et al., 2004*; Lefaucheur et al., 2006*; Jetté et al., 2013*				Mixed
8	5 Hz, long sessions versus sham rTMS	rTMS	5 Hz	15 sessions	Sham rTMS	PHN	VAS	Moisset et al., 2020b	Pei et al., 2019		2	0/0/1/2	Potentially better
		rTMS	5 Hz	12 sessions	Sham rTMS	CPSP	VAS	Kumru et al., 2017; Ramger et al., 2019^&^; Yang and Chang, 2020		Kobayashi et al., 2015 ^&^			Potentially better (up to 8 weeks)
9	5 Hz, medium sessions versus sham rTMS	rTMS	5 Hz	10 sessions	Sham rTMS	SCI, mixed NP, CPSP, BPL, PLP, TGNP, and PNI	VAS	Zaghi et al., 2011; Mehta et al., 2013; Boldt et al., 2014^#^; Moreno-Duarte et al., 2014; Galhardoni et al., 2015; Jin et al., 2015; Mulla et al., 2015; Chen et al., 2016; Cragg et al., 2016; Gao et al., 2017; Kumru et al., 2017; Herrero Babiloni et al., 2018; O’Connell et al., 2018*; Gatzinsky et al., 2020; Moisset et al., 2020b; Shen et al., 2020; Yang and Chang, 2020; Yu et al., 2020	Defrin et al., 2007*^#^; Hosomi et al., 2013*		2	2/1/0/2	Mixed
10	5 Hz, short sessions versus sham rTMS	rTMS	5 Hz	5 sessions	Sham rTMS	Mixed NP	VAS	Hamid et al., 2019; Liampas et al., 2020a; Yang and Chang, 2020	Shimizu et al., 2017		4	3/0/1/4	Potentially better
		rTMS	5 Hz	3 sessions	Sham rTMS	CPSP,SCI, PL, BPL, and PNI	VAS	Jin et al., 2015; Gao et al., 2017; Kumru et al., 2017; O’Connell et al., 2018*; Yang and Chang, 2020	Saitoh et al., 2007 *				Potentially better
		rTMS	5 Hz	1 session	Sham rTMS	CPSP	VAS or NRS	Galhardoni et al., 2015; Jin et al., 2015; Kumru et al., 2017; Hamid et al., 2019; Ramger et al., 2019^&^; Yang and Chang, 2020	Hosomi et al., 2013*; Matsumura et al., 2013*^&^				Potentially better
		rTMS	5 Hz	1 session	Sham rTMS	PLP	VAS or NRS	Galhardoni et al., 2015; O’Connell et al., 2018*; Pacheco-Barrios et al., 2020	Irlbacher et al., 2006 *			1/0/0/1	No difference
11	1 Hz, short sessions versus sham rTMS	rTMS	1 Hz	3 sessions	Sham rTMS	Mixed NP, CPSP, SCI, PNP, PLP, BPL, and TGNP	VAS, NRS	Leung et al., 2009; Zaghi et al., 2011; Galhardoni et al., 2015; Gao et al., 2017; Kumru et al., 2017; O’Connell et al., 2018*; Yang and Chang, 2020	Andre-Obadia et al., 2006*; Saitoh et al., 2007*; Lefaucheur et al., 2008*		4	4/0/0/4	No difference
		rTMS	1 Hz	1 session	Sham rTMS	PLP	VAS orNRS	Galhardoni et al., 2015; O’Connell et al., 2018*; Pacheco-Barrios et al., 2020	Irlbacher et al., 2006 *				No difference
12	0.5 Hz, short sessions versus sham rTMS	rTMS	0.5 Hz	3 sessions	Sham rTMS	Mixed NP, CPSP, and BPL	VAS	Zaghi et al., 2011; Galhardoni et al., 2015; Jin et al., 2015; Kumru et al., 2017; Yang and Chang, 2020	Lefaucheur et al., 2001b		1	0/0/0/1	Mixed
13	rTMS on M1 versusrTMS on S1, SMA, PMC, and sham	rTMS on M1 area	5 Hz	2 sessions	rTMS on S1,SMA,PMC area	CPSP, SCI, TGNI, PNI, and RA	VAS	Kumru et al., 2017	Saitoh et al., 2006		1	0/0/0/1	Potentially better
		rTMS on M1 area	5 Hz	4 sessions	rTMS on S2,SMA,PMC area	Mixed NP, CPSP, SCI, TGNP, and PNP	VAS	Leung et al., 2009; Galhardoni et al., 2015; Jin et al., 2015; Chen et al., 2016; Kumru et al., 2017; O’Connell et al., 2018*; Gatzinsky et al., 2020; Yang and Chang, 2020	Hirayama et al., 2006 *		1	1/0/0/1	Mixed
14	bilateral M1 versus unilateral M1	rTMS:bilateral M1	10 Hz	1 session	rTMS, unilateral M1	TN		Yang and Chang, 2020	Henssen et al., 2019		1	0/0/0/1	Potentially better
15	Single rTMS versus 5 rTMS sessions	rTMS	10 Hz	1 session	rTMS, 5 sessions	CRPS	VAS, NRS	Chang et al., 2020; Yang and Chang, 2020		Gaertner et al., 2018	1	0/0/0/1	Mixed
16	3 true rTMS versus 1 true + 2 sham rTMS	rTMS	10 Hz	3 sessions true	1 true sessions and 2 sham sessions	Migraine	VAS	Stilling et al., 2019		Misra et al., 2017	1	0/0/0/1	Potentially better
		rTMS	10 Hz	3 sessions true	1 true sessions and 2 sham sessions	Migraine	VAS	Stilling et al., 2019; Moisset et al., 2020a	Kalita et al., 2016		1	0/0/0/1	No difference
17	rTMS versus BTX-A Injection	rTMS	10 Hz	12 sessions	BTX-A injection	Migraine		Yang and Chang, 2020	Shehata et al., 2016		1	0/0/0/1	Unclear
		rTMS	10 Hz	12 sessions	BTX-A 31 to 39 sites	Migraine	VAS	Stilling et al., 2019; Moisset et al., 2020a	Shehata et al., 2016		1	0/0/0/1	No difference
		rTMS	10 Hz	12 sessions	BTX-A 31 to 39 sites	Migraine	VAS	Stilling et al., 2019; Moisset et al., 2020a	Shehata et al., 2016		1	0/0/0/1	Potentially worse (at week 12)
18	rTMS + tDCS versussham rTMS and tDCS	rTMS and tDCS	10 Hz	3 sessions	Sham rTMS and2-mA tDCS	Radiculopathy	NRS	O’Connell et al., 2018; Aamir et al., 2020; Gatzinsky et al., 2020; Yang and Chang, 2020*	Attal et al., 2016 *		1	1/0/0/1	Mixed
19	rTMS + TBS versus rTMS	rTMS+ TBS	10 Hz	1 session	rTMS	TGNP, CPSP, and SCI	VAS	Galhardoni et al., 2015; Kumru et al., 2017	Lefaucheur et al., 2012		1	0/0/0/1	Potentially better

*PICO, population, intervention, control group, outcome; RCT, Randomized controlled trial; rTMS, Repetitive transcranial magnetic stimulation; M1, Motor cortex; S1, Primary somatosensory cortex; SMA, Supplementary motor cortex; PMC, Premotor cortex; BTX-A, Botulinum toxin type A; tDCS, Transcranial direct current stimulation; TBS, Theta-burst stimulation; OFP, Orofacial pain; NP, Neuropathic pain; LBP, Low back pain; TN, Trigeminal neuralgia; CPSP, Central post-stroke pain; PLP, Phantom limb pain; MBTI-HA, Mild traumatic brain injury related headache; NTL, Nerve trunk lesion; TGNP, Trigeminal neuropathic pain; IBS, Irritable bowel syndrome; SCI, Spinal cord injury; BPL, Brachial plexus lesion; DPN, Diabetic peripheral neuropathy; PHN, Postherpetic neuralgia; PNI, peripheral nerve injury; PNP, peripheral neuropathic pain; TGNI, trigeminal nerve injury; RA, root avulsion; VAS, Visual analog scale; NRS, Numerical rating scale.*

***Notes:** short, 1–5 sessions, medium, 5–10 sessions, long, >10 sessions.*

In the [Included SRs], high-quality SRs are marked as *, moderate quality SRs are marked as #, low-quality SRs are marked as &, and the rest are critically-low-quality SRs.

In the [Primary studies included in SRs], *, included by high and critically-low-quality SRs; **, Only included by high-quality SRs; #, Included by moderate and critically-low-quality SRs; &, Included by low and critically-low-quality SRs.

*In the [Number of SRs involving the quality (high/moderate/low/critically low) of primary studies], Taking the 9th PICOs (5 Hz, medium sessions vs. sham rTMS), as an example, a total of 2 primary studies were involved. The meaning of 2/1/0/2 is shown in Figure 4.*

The key characteristics of PICOs are listed in Supplementary Material 5. A total of 19 PICOs were categorized based on stimulation target, frequency, and session (short, medium, and long). On the basis of the stimulation target, 17 PICOs used high-frequency rTMS (>1 Hz), and 2 used low-frequency rTMS (<1 Hz). In terms of the number of sessions, 1–5 sessions were considered short sessions, 6–10 were medium sessions, and more than 10 were regarded as long sessions. Twelve PICOs had short sessions, three had medium sessions, and four had long sessions. In addition, 12 PICOs used the same sessions of sham stimulation or placebo as a control to study the effectiveness of rTMS in patients with NP. Two PICOs studied the effects of different sessions of rTMS, while other PICOs involved different stimulation areas: M1 unilateral stimulation versus bilateral stimulation, rTMS compared with botulinum toxin injection, rTMS compared with transcranial direct current stimulation (tDCS), or rTMS combined with theta-burst stimulation. The PICOs were concentrated in the following characteristics: 20 Hz, short-term sessions versus sham stimulation (11 PICOs), and 10 Hz, short-term sessions versus sham stimulation (18 PICOs).

### Specific Findings From Systematic Reviews in Evidence Mapping

**FIGURE 5:**
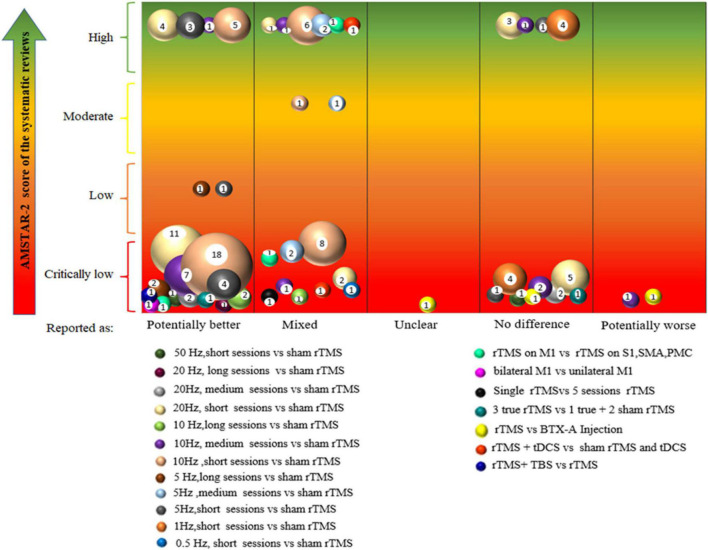
Evidence mapping of the rTMS on neuropathic pain (NP). short, 1–5 sessions, medium, 5–10 sessions, long, >10 sessions. rTMS, repetitive transcranial magnetic stimulation; tDCS, transcranial direct current stimulation; TBS, theta-burst stimulation; M1,motor cortex, S1, primary somatosensory cortex; SMA, supplementary motor cortex; PMC, premotor cortex; BTX-A, botulinum toxin type A.

The evidence map of rTMS for NP is presented in Figure 5. The bubble diagram is a visual display of data represented in Supplementary Material 5. We integrated similar intervention characteristics from primary studies into PICOs. In the bubble chart, different colors indicate varying PICOs. Each bubble was plotted in accordance with the conclusion of the effect of rTMS on NP (X-axis) and the quality of the related SRs (Y-axis), while the size of bubbles represented the number of primary studies included in PICOs. The evidence tables (Supplementary Material 5) provided details of the included SRs (Supplementary Material 4). Some primary studies may be included in multiple SRs. If SRs synthesized different conclusions for the same primary study, the same PICOs would appear in different classifications on the X-axis. If the same primary study was included by SRs of different quality, the same PICOs would appear in different classifications on the Y-axis. Evidence mapping showed that 5–20 Hz, high-frequency rTMS of M1 with short (1–5), medium (6–10), or long (>10) sessions usually lead to “potentially better” treatment effects compared with sham stimulation, although some had transient effects. By contrast, the synthesis results for the lower frequencies (1 and 0.5 Hz) showed either no difference or mixed effects. Thirteen PICOs included 52 primary studies rated as “potentially better,” and four of these PICOs involved 13 primary studies that were also included in a high-quality meta-analysis. In accordance with the AMSTAR-2 quality assessment, the interventions in these four PICOs were considered beneficial in most cases. Nine PICOs included 18 primary studies with different findings in different SRs and were rated as “mixed,” implying that the interventions in these eight PICOs had limited confidence in the effect estimates, and the true effect may be different from the study reports (Miake-Lye et al., 2019). One PICO conclusion was rated as “unclear” because its effect was not reported in the SR (Yang and Chang, 2020) with a critically low quality. Eight PICOs included 17 primary studies that concluded that rTMS showed no difference compared with controls. Of these, six studies showed a potentially better effect of rTMS in short-term follow-up but no difference during long-term follow up (Supplementary Material 4 and Supplementary Material 5). After studies that were ineffective during follow-up were excluded, 8 of 11 primary studies were also included by a high-quality meta-analysis. This finding indicated less effectiveness of these intervention protocols or inapplicability to a particular NP, and the treatment effects could be uncertain. Two PICOs included two primary studies that showed a “potentially worse” conclusion.

## Discussion

### Principal Findings of Evidence Mapping

This evidence map included 40 SRs, and the majority of the primary studies included were RCTs, which is the best study design to assess the effectiveness of interventions (Sylvester et al., 2017). Evidence mapping provided a broad overview of the available evidence of rTMS on NP, showing the focus and counting contributions of available studies by categorizing and generalizing them to help interpret the published SRs. (1) Research gaps: The included SRs covered most types of NP, including SCI, CPSP, CRPS, PLP, DNP, and headache; however, this left an evidence gap of the rTMS for some specific types of NP, such as postherpetic pain, radiculopathy pain, trigeminal neuralgia, post-traumatic brain injury pain, and cancer-related NP. In addition, the control groups were mostly given sham stimulation. Open questions about the effectiveness of rTMS associated with other therapies (such as pharmacotherapy, neurorehabilitation, and psychotherapy) are recommended. Future SRs are needed to analyze immediate, short-term, and long-term effects, which may help clarify the sessions of rTMS. Stimulation parameters, namely, frequency and intensity variable time, are also the direction for further research. (2) Summarization and classification of evidence: Evidence mapping showed that 5–20 Hz, high-frequency rTMS of M1 with short (1–5), medium (6–10), or long (>10) sessions usually lead to a “potentially better” conclusion compared with sham stimulation, suggesting that these interventions are beneficial in most cases. By contrast, the synthesis results for the low frequencies (1 and 0.5 Hz) showed no difference or were mixed, meaning these intervention protocols may be less effective or inappropriate for some specific NPs. (3) The impact of the quality of the SRs on the strength of evidence: Some PICOs from high-quality SRs drew a potentially better conclusion, suggesting that these interventions were beneficial in most cases. Similarly, some PICOs from high-quality SRs did not show any difference in the conclusion, indicating that these interventions may be less effective or inappropriate for some specific NPs. This evidence map is not intended to replace any clinical protocol or guidelines, nor is it intended to provide a standardized protocol. Therefore, the clinical diagnosis of each patient, the existing alternatives, cost-effectiveness, available resources, and other factors must be carefully considered before offering any recommendation (Nussbaumer-Streit et al., 2018).

### Comparison of Results of Systematic Reviews

By comparing the results of the SRs, the same PICOs obtained from SRs were presented with different conclusions in the evidence map. The possible reasons were as follows: (1) some studies that reported different sessions had varied effects of rTMS. For example, a good outcome could be found in the short term, but not in the long term, that led to a mixed conclusion. Future SRs should focus on follow-up and explore the long-term effectiveness of the intervention. (2) Some SRs conducted meta-analysis but some did not. Qualitative studies may arrive at conclusions different from those in quantitative studies. For example, a primary study by Onesti et al. (2013) and meta-analysis of Zeng et al. (2020) had a “no difference” conclusion, while the review of Yang and Chang (2020), which only mentioned pain relief, showed a “potentially better” conclusion. The final conclusion in the evidence map of Onesti et al.’s study was “mixed.” (3) Populations with different diseases that cause NP could cause heterogeneity. Previous studies have indicated that patients with orofacial pain have a better analgesic response than those with CPSP, SCI, or BPL (Lefaucheur et al., 2004). For example, a primary study on patients with CRPS (Picarelli et al., 2010) by Chang et al. (2020) and another one involving chronic pain by O’Connell et al. (2018) did not reach the same conclusion. This gap could also be a caveat to provide more reliable and reproducible data. Future studies must consider including more homogeneous groups of participants or stratifying patients in accordance with clinical characteristics and underlying pathogenesis.

If different SRs with varied conclusions included some primary studies that were overlapping and some unique studies, future investigations could synthesize studies that were included in these reviews and find new outcomes including all potential evidence. From the “mixed” results, future investigations could focus on comparing different stimulation protocols (doses, sessions, variable time, and intervals among sessions) of rTMS on NP.

### Overall Completeness and Validity of Evidence Mapping

We evaluated the quality of SRs and the credibility of the results of SRs with a new version of AMSTAR. Compared with the previous version, AMSTAR-2 was adapted to include SRs on the basis of RCTs or non-randomized intervention studies or both and provide more refined and rigorous evaluation item criteria. Assessment in this field suggested room for improving SRs’ quality. Future SRs should place more emphasis on the following domains to improve the quality of studies and the validity of the results: reporting an explicit statement about the description of the methodology prior to conducting the review; any significant deviations from the protocol should be justified; explaining the selection of the study designs for inclusion in the review; providing a list of excluded studies and justifying the exclusions; indicating the sources of funding or support for the individual studies included in the SRs; and interpreting or discussing the effect of the risk of bias in individual studies on the total effect.

### Strengths and Limitations of This Evidence Mapping

This evidence map may be the first on rTMS for NP. Evidence mapping is a relatively new tool for the synthesis of available evidence, so we explain the methodology and results in more detail compared with published evidence maps and provide all the data of our study process to facilitate the reader’s understanding and use. To avoid selection and data extraction bias, we constructed a rich search string for retrieving from four different databases. In addition, the reference lists of the selected studies were manually scanned for the detection of additional relevant studies, minimizing the risk of missing relevant studies. Study selection and data extraction were made via a double confirmation. Two authors conducted the process of selecting and extracting separately from one another, and any disagreements were then discussed with a third researcher until a final agreement was reached. Furthermore, results were mapped using various graphs, such as bubble plots, heat maps, and tables, which helped improve traceability between the extracted data and the conclusion.

Certain limitations in this evidence map should be considered. First, the map only chose studies published in English, which limited the scope of evidence mapping. Second, given that only SRs were included as a source of evidence, some studies, such as newly published or studies included in these SRs, could have been missed. Third, the methodologies of some SRs had limitations. Furthermore, several different types of studies in SRs comparing therapeutic interventions for NP were included. Although most trials were RCTs, some case reports and observational, open-label, and cohort studies were also available. Finally, evidence mapping synthesized the SRs as a unit rather than individual studies, which could lead to some primary studies being repeated.

## Conclusion

Neuropathic pain is a complex and refractory group of diseases. Evidence mapping showed that rTMS, as a compliant and safety neuromodulation treatment, is promising for the treatment of NP. Evidence mapping could encourage clinicians and professionals involved in related areas, such as pain, neurology, psychiatry, and anesthesiology, to pay more attention to non-pharmacological treatments on patients with NPs, especially those with drug resistance. Evidence mapping is a useful and reliable method to identify the currently available evidence on therapeutic interventions and pinpoint gaps to suggest future research. In the future, when designing treatment protocols, rehabilitation practitioners are recommended to consider the duration and sessions of rTMS. More research efforts are needed to highlight the optimal stimulation protocols and standardize all parameters to fill evidence gaps, and more homogeneous groups of participants should be considered. Meanwhile, as the methodological quality of most included SRs scored “critically low,” further efforts are needed to improve the methodological quality and reporting process of SRs.

## Data Availability Statement

The original contributions presented in the study are included in the article/Supplementary Material, further inquiries can be directed to the corresponding author.

## Author Contributions

YZa, YoZ, and YiZ were designed the study. YZa and XL were collected the screening studies, extraction data and charted under the guidance of YoZ. YZa and YoZ analyzed the data and drafted the manuscript. YiZ reviewed the results. SG, YY, JG, and YiZ revised the manuscript for important intellectual content. All authors approved the final version of the manuscript.

## Conflict of Interest

The authors declare that the research was conducted in the absence of any commercial or financial relationships that could be construed as a potential conflict of interest.

## Publisher’s Note

All claims expressed in this article are solely those of the authors and do not necessarily represent those of their affiliated organizations, or those of the publisher, the editors and the reviewers. Any product that may be evaluated in this article, or claim that may be made by its manufacturer, is not guaranteed or endorsed by the publisher.
