# Supplementary material for: Evidence Mapping Based on Systematic Reviews of Repetitive Transcranial Magnetic Stimulation on the Motor Cortex for Neuropathic Pain
Source: Front Hum Neurosci. 2022 Feb 16;15:743846. doi: 10.3389/fnhum.2021.743846 (PMC8889530; doi:10.3389/fnhum.2021.743846)
Supplement: Supplementary file 1 [file Data_Sheet_1.zip › Supplementary Material Files/Supplementary material 2 SRs excluded.docx]

**Supplementary material 2:**

**studies that were evaluated in full-text and were excluded.**

| **N** | **Author** | **Year** | **Title** | **Reason for exclusion** |
| --- | --- | --- | --- | --- |
| 1 | Cassani, R. et al | 2020 | Virtual reality and non-invasive brain stimulation for rehabilitation applications: a systematic review | Non-interested population:  TMS is not associated with neuropathic pain in the population treated |
| 2 | Young, J. R. et al | 2020 | Non-invasive brain stimulation modalities for the treatment and prevention of opioid use disorder: a systematic review of the literature | Non-interested population:  study on opioid use disorder; |
| 3 | Xu, X. M. . et al | 2020 | Nonpharmacological therapies for central poststroke pain: A systematic review | Non-interested site |
| 4 | Reuter, U. et al | 2019 | Non-invasive neuromodulation for migraine and cluster headache: a systematic review of clinical trials | Non-interested site |
| 5 | Treister, A. K et al | 2017 | Demystifying Poststroke Pain: From Etiology to Treatment | No appropriate control group |
| 6 | Di Pietro, F.et al | 2013 | Primary motor cortex function in complex regional pain syndrome: A systematic review and meta-analysis | No pain outcome:  Study Assesses Changes in Primary Motor Cortex in Complex Regional Pain Syndrome by Several Neuroimaging Techniques |
| 7 | Nardone, R. et al | 2018 | Transcranial magnetic stimulation studies in complex regional pain syndrome type I: A review | No pain outcome  Study NP-related pathophysiology |
| 8 | Reuter, U. et al | 2019 | Non-invasive neuromodulation for migraine and cluster headache: a systematic review of clinical trials | No pain outcome:  aim to assess the scientific rigour and clinical relevance of these  devices and their associated clinical data. |
| 9 | Nardone, R. et al | 2015 | Descending motor pathways and cortical physiology after spinal cord injury assessed by transcranial magnetic stimulation: a systematic review | No pain outcome  This study aim to identify neurophysiological biomarkers through TMS technology to help assess the extent of nerve damage, elucidate mechanisms of nerve repair, predict clinical outcomes, and identify therapeutic targets. |
| 10 | Parker, R. S. et al | 2016 | Is Motor Cortical Excitability Altered in People with Chronic Pain? A Systematic Review and Meta-Analysis | No pain outcome  This review studies examining corticospinal and intracortical  excitability using transcranial magnetic stimulation in people with chronic pain compared to healthy controls |
| 11 | Araújo, H. A. et al | 2011 | Systematic literature review on the effects of noninvasive cortical stimulation for chronic pain control | Non-interested study design ；Conference abstract |
| 12 | Cruccu, G. et al | 2007 | EFNS guidelines on neurostimulation therapy for neuropathic pain | Non-interested study design ；guideline |
| 13 | Cruccu, G. et al | 2016 | EAN guidelines on central neurostimulation therapy in chronic pain conditions | Non-interested study design ；guideline |
| 14 | Dosenovic, S. et al | 2017 | Interventions for Neuropathic Pain: An Overview of Systematic Reviews | Non-interested study design；  An overview of the study of systematic reviews |
| 15 | Soulia, Vassiliki et al | 2011 | Non-invasive and Non-pharmacological Methods for the Alleviation of Neuropathic Pain | Non-English |
