# Supplementary material for: Evidence Mapping Based on Systematic Reviews of Repetitive Transcranial Magnetic Stimulation on the Motor Cortex for Neuropathic Pain
Source: Front Hum Neurosci. 2022 Feb 16;15:743846. doi: 10.3389/fnhum.2021.743846 (PMC8889530; doi:10.3389/fnhum.2021.743846)
Supplement: Supplementary file 1 [file Data_Sheet_1.zip › Supplementary Material Files/Supplementary Material 5.docx]

**Included systematic review (meta analysis)**

[1] C. Zucchella and E. Mantovani and R. De Icco et al. "Non-invasive Brain and Spinal Stimulation for Pain and Related Symptoms in Multiple Sclerosis: A Systematic Review". Frontiers in Neuroscience 2020;14.

[2] H. Zeng and K. Pacheco-Barrios and Y. Cao et al. "Non-invasive neuromodulation effects on painful diabetic peripheral neuropathy: a systematic review and meta-analysis". Sci Rep 2020;10:19184.

[3] B. Yu and H. Qiu and J. Li and C. Zhong, J. Li "Noninvasive Brain Stimulation Does Not Improve Neuropathic Pain in Individuals With Spinal Cord Injury: Evidence From a Meta-Analysis of 11 Randomized Controlled Trials". Am J Phys Med Rehabil 2020;99:811-820.

[4] S. Yang, M.C. Chang "Effect of Repetitive Transcranial Magnetic Stimulation on Pain Management: A Systematic Narrative Review". Front Neurol 2020;11:114.

[5] Z. Shen and Z. Li and J. Ke et al. "Effect of non-invasive brain stimulation on neuropathic pain following spinal cord injury: A systematic review and meta-analysis". Medicine (Baltimore) 2020;99:e21507.

[6] K. Pacheco-Barrios ,X. Meng,F. Fregni "Neuromodulation Techniques in Phantom Limb Pain: A Systematic Review and Meta-analysis". Pain Med 2020;21:2310-2322.

[7] X. Moisset and B. Pereira and D. Ciampi de Andrade et al. "Neuromodulation techniques for acute and preventive migraine treatment: a systematic review and meta-analysis of randomized controlled trials". J Headache Pain 2020;21:142.

[8] X. Moisset and D. Bouhassira and J. Avez Couturier et al. "Pharmacological and non-pharmacological treatments for neuropathic pain: Systematic review and French recommendations". Rev Neurol (Paris) 2020;176:325-352.

[9] A. Liampas and N. Velidakis and T. Georgiou et al. "Prevalence and Management Challenges in Central Post-Stroke Neuropathic Pain: A Systematic Review and Meta-analysis". Advances in Therapy 2020;37:3278-3291.

[10] A. Liampas and M. Rekatsina and A. Vadalouca et al. "Non-Pharmacological Management of Painful Peripheral Neuropathies: A Systematic Review". Adv Ther 2020;37:4096-4106.

[11] K. Gatzinsky and C. Bergh and A. Liljegren et al. "Repetitive transcranial magnetic stimulation of the primary motor cortex in management of chronic neuropathic pain: a systematic review". Scand J Pain 2020.

[12] M.C. Chang ,S.G. Kwak,D. Park "The effect of rTMS in the management of pain associated with CRPS". Translational Neuroscience 2020;11:363-370.

[13] A. Cardenas-Rojas and K. Pacheco-Barrios and S. Giannoni-Luza and O. Rivera-Torrejon, F. Fregni "Noninvasive brain stimulation combined with exercise in chronic pain: a systematic review and meta-analysis". Expert Review of Neurotherapeutics 2020;20:401-412.

[14] A. Aamir and A. Girach and P.G. Sarrigiannis et al. "Repetitive Magnetic Stimulation for the Management of Peripheral Neuropathic Pain: A Systematic Review". Adv Ther 2020;37:998-1012.

[15] J.M. Stilling and O. Monchi and F. Amoozegar, C.T. Debert "Transcranial Magnetic and Direct Current Stimulation (TMS/tDCS) for the Treatment of Headache: A Systematic Review". Headache 2019;59:339-357.

[16] B.C. Ramger and K.A. Bader and S.P. Davies et al. "Effects of non-invasive brain stimulation on clinical pain intensity and experimental pain sensitivity among individuals with central post-stroke pain: A systematic review". Journal of Pain Research 2019;12:3319-3329.

[17] R. Nardone and V. Versace and L. Sebastianelli et al. "Transcranial magnetic stimulation in subjects with phantom pain and non-painful phantom sensations: A systematic review". Brain Res Bull 2019;148:1-9.

[18] P. Hamid ,B.H. Malik,M.L. Hussain "Noninvasive Transcranial Magnetic Stimulation (TMS) in Chronic Refractory Pain: A Systematic Review". Cureus 2019;11:e6019.

[19] Y. Feng and B. Zhang and J. Zhang, Y. Yin "Effects of Non-invasive Brain Stimulation on Headache Intensity and Frequency of Headache Attacks in Patients With Migraine: A Systematic Review and Meta-Analysis". Headache 2019;59:1436-1447.

[20] G. Akyuz, E. Giray "Noninvasive neuromodulation techniques for the management of phantom limb pain: a systematic review of randomized controlled trials". Int J Rehabil Res 2019;42:1-10.

[21] N.E. O'Connell and L. Marston and S. Spencer and L.H. Desouza, B.M. Wand "Non-invasive brain stimulation techniques for chronic pain". Cochrane Database of Systematic Reviews 2018;2018.

[22] A. Herrero Babiloni and S. Guay and D.R. Nixdorf and L. de Beaumont, G. Lavigne "Non-invasive brain stimulation in chronic orofacial pain: a systematic review". J Pain Res 2018;11:1445-1457.

[23] L. Lan and X. Zhang and X. Li and X. Rong, Y. Peng "The efficacy of transcranial magnetic stimulation on migraine: a meta-analysis of randomized controlled trails". J Headache Pain 2017;18:86.

[24] H. Kumru and S. Albu and J. Vidal, J.M. Tormos "Effectiveness of repetitive trancranial or peripheral magnetic stimulation in neuropathic pain". Disabil Rehabil 2017;39:856-866.

[25] B. Goudra and D. Shah and G. Balu et al. "Repetitive Transcranial Magnetic Stimulation in Chronic Pain: A Meta-analysis". Anesth Essays Res 2017;11:751-757.

[26] F. Gao and H. Chu and J. Li et al. "Repetitive transcranial magnetic stimulation for pain after spinal cord injury: a systematic review and meta-analysis". J Neurosurg Sci 2017;61:514-522.

[27] J.J. Cragg and F.M. Warner and N.B. Finnerup et al. "Meta-analysis of placebo responses in central neuropathic pain: Impact of subject, study, and pain characteristics". Pain 2016;157:530-540.

[28] C.C. Chen and Y.F. Chuang and A.C. Huang and C.K. Chen, Y.J. Chang "The antalgic effects of non-invasive physical modalities on central post-stroke pain: a systematic review". J Phys Ther Sci 2016;28:1368-1373.

[29] S.M. Mulla and L. Wang and R. Khokhar et al. "Management of Central Poststroke Pain: Systematic Review of Randomized Controlled Trials". Stroke 2015;46:2853-2860.

[30] Y. Jin and G. Xing and G. Li et al. "High Frequency Repetitive Transcranial Magnetic Stimulation Therapy For Chronic Neuropathic Pain: A Meta-analysis". Pain Physician 2015;18:E1029-1046.

[31] R. Galhardoni and G.S. Correia and H. Araujo et al. "Repetitive transcranial magnetic stimulation in chronic pain: a review of the literature". Arch Phys Med Rehabil 2015;96:S156-172.

[32] I. Moreno-Duarte and L.R. Morse and M. Alam et al. "Targeted therapies using electrical and magnetic neural stimulation for the treatment of chronic pain in spinal cord injury". NeuroImage 2014;85:1003-1013.

[33] I. Boldt and I. Eriks‐Hoogland and M.W.G. Brinkhof et al. "Non‐pharmacological interventions for chronic pain in people with spinal cord injury". Cochrane Database of Systematic Reviews 2014.

[34] S. Mehta and K. Orenczuk and A. McIntyre et al. "Neuropathic pain post spinal cord injury part 1: systematic review of physical and behavioral treatment". Top Spinal Cord Inj Rehabil 2013;19:61-77.

[35] L. Cossins and R.W. Okell and H. Cameron et al. "Treatment of complex regional pain syndrome in adults: a systematic review of randomized controlled trials published from June 2000 to February 2012". Eur J Pain 2013;17:158-173.

[36] S. Zaghi and B. Thiele and D. Pimentel and T. Pimentel, F. Fregni "Assessment and treatment of pain with non-invasive cortical stimulation". Restorative Neurology and Neuroscience 2011;29:439-451.

[37] A. Leung and M. Donohue and R. Xu et al. "rTMS for suppressing neuropathic pain: a meta-analysis". J Pain 2009;10:1205-1216.

[38] B. Kumar and J. Kalita and G. Kumar, U.K. Misra "Central poststroke pain: A review of pathophysiology and treatment". Anesthesia and Analgesia 2009;108:1645-1657.

**Primary studies included in the SRs**

[1] Seada Y. I., Nofel R., Sayed H. M. (2013). Comparison between trans-cranial electromagnetic stimulation and low-level laser on modulation of trigeminal neuralgia. J. Phys. Ther. Sci. 25, 91–914.

[2] Korzhova J., Bakulin I., Sinitsyn D., Poydasheva A., Suponeva N., Zakharova M., et al. . (2019). High-frequency repetitive transcranial magnetic stimulation and intermittent theta-burst stimulation for spasticity management in secondary progressive multiple sclerosis. Eur. J. Neurol. 26, 680–e44.

[3] Onesti, E. et al. H-coil repetitive transcranial magnetic stimulation for pain relief in patients with diabetic neuropathy. Eur. J. Pain (Lond. Engl.) 17, 1347–1356.

[4] Seada Y. I., Nofel R., Sayed H. M. (2013). Comparison between trans-cranial electromagnetic stimulation and low-level laser on modulation of trigeminal neuralgia. J. Phys. Ther. Sci. 25, 91–914.

[5] Abdelkader, A. A., El Gohary, A. M. & Mourad, H. S. Repetitive TMS in treatment of resistant diabetic neuropathic pain. Egypt. J. Neurol. Psychiatry Neurosurg. 55, 1 (2019).

[6] Defrin R, Grunhaus L, Zamir D, et al: The effect of a series of repetitive transcranial magnetic stimulations of the motor cortex on central pain after spinal cord injury. Arch Phys Med Rehabil 2007;88:1574–80.

[7] Kang BS, Shin HI, Bang MS: Effect of repetitive transcranial magnetic stimulation over the hand motor cortical area on central pain after spinal cord injury. Arch Phys Med Rehabil 2009;90:1766–71.

[8] Yílmaz B, Kesikburun S, Yaşar E, et al: The effect of repetitive transcranial magnetic stimulation on refractory neuropathic pain in spinal cord injury. J Spinal Cord Med 2014;37:397–400.

[9] Lefaucheur JP, Drouot X, Keravel Y, Nguyen JP. Pain relief induced by repetitive transcranial magnetic stimulation of precentral cortex. Neuroreport. (2001) 12:2963–5.

[10] Rollnik JD, Wustefeld S, Dauper J, Karst M, Fink M, Kossev A, et al. . Repetitive transcranial magnetic stimulation for the treatment of chronic pain – a pilot study. Eur Neurol. (2002) 48:6–10.

[11] Lefaucheur JP, Drouot X, Menard-Lefaucheur I, Nguyen JP. Neuropathic pain controlled for more than a year by monthly sessions of repetitive transcranial magnetic stimulation of the motor cortex. Neurophysiol Clin. (2004) 34:91–5.

[12] Andre-Obadia N, Peyron R, Mertens P, Mauguière F, Laurent B, Garcia-Larrea L. Transcranial magnetic stimulation for pain control. Double-blind study of different frequencies against placebo, and correlation with motor cortex stimulation efficacy. Clin Neurophysiol. (2006) 117:1536–44.

[13] Hirayama A, Saitoh Y, Kishima H, Shimokawa T, Oshino S, Hirata M, et al. . Reduction of intractable deafferentation pain by navigation-guided repetitive transcranial magnetic stimulation of the primary motor cortex. Pain. (2006) 122:22–7.

[14] Lefaucheur JP, Hatem S, Nineb A, Ménard-Lefaucheur I, Wendling S, Keravel Y, et al. . Somatotopic organization of the analgesic effects of motor cortex rTMS in neuropathic pain. Neurology. (2006) 67:1998–2004.

[15] Lefaucheur JP, Drouot X, Menard-Lefaucheur I, Keravel Y, Nguyen JP. Motor cortex rTMS in chronic neuropathic pain: pain relief is associated with thermal sensory perception improvement. J Neurol Neurosurg Psychiatr. (2008) 79:1044–9.

[16] Andre-Obadia N, Mertens P, Gueguen A, Peyron R, Garcia-Larrea L. Pain relief by rTMS: differential effect of current flow but no specific action on pain subtypes. Neurology. (2008) 71:833–40.

[17] Lefaucheur JP, Ayache SS, Sorel M, Farhat WH, Zouari HG, Ciampi de Andrade D, et al. . Analgesic effects of repetitive transcranial magnetic stimulation of the motor cortex in neuropathic pain: influence of theta burst stimulation priming. Eur J Pain. (2012) 16:1403–13.

[18] Hosomi K, Shimokawa T, Ikoma K, Nakamura Y, Sugiyama K, Ugawa Y, et al. . Daily repetitive transcranial magnetic stimulation of primary motor cortex for neuropathic pain: a randomized, multicenter, double-blind, crossover, sham-controlled trial. Pain. (2013) 154:1065–72.

[19] Khedr EM, Kotb HI, Mostafa MG, Mohamad MF, Amr SA, Ahmed MA, et al. . Repetitive transcranial magnetic stimulation in neuropathic pain secondary to malignancy: a randomized clinical trial. Eur J Pain. (2014) 19:519–27.

[20] Attal N, Ayache SS, Ciampi De Andrade D, Mhalla A, Baudic S, Jazat F, et al. . Repetitive transcranial magnetic stimulation and transcranial direct-current stimulation in neuropathic pain due to radiculopathy: a randomized sham-controlled comparative study. Pain. (2016) 157:1224–31.

[21] Ayache SS, Ahdab R, Chalah MA, Farhat WH, Mylius V, Goujon C, et al. . Analgesic effects of navigated motor cortex rTMS in patients with chronic neuropathic pain. Eur J Pain. (2016) 20:1413–22.

[22] Nurmikko T, MacIver K, Bresnahan R, Hird E, Nelson A, Sacco P. Motor cortex reorganization and repetitive transcranial magnetic stimulation for pain-a methodological study. Neuromodulation. (2016) 19:669–78.

[23] Pommier B, Creac’h C, Beauvieux V, Nuti C, Vassal F, Peyron R. Robot-guided neuronavigated rTMS as an alternative therapy for central. (neuropathic) pain: clinical experience and long-term follow-up. Eur J Pain. (2016) 20:907–16.

[24] Shimizu T, Hosomi K, Maruo T, Goto Y, Yokoe M, Kageyama Y, et al. . Efficacy of deep rTMS for neuropathic pain in the lower limb: a randomized, double-blind crossover trial of an H-coil and figure-8 coil. J Neurosurg. (2017) 127:1172–80.

[25] Andre-Obadia N, Magnin M, Simon E, Garcia-Larrea L. Somatotopic effects of rTMS in neuropathic pain? A comparison between stimulation over hand and face motor areas. Eur J Pain. (2018) 22:707–15.

[26] Lawson McLean A, Frank S, Zafar N, Waschke A, Kalff R, Reichart R. Time course of the response to navigated repetitive transcranial magnetic stimulation at 10 Hz in chronic neuropathic pain. Neurol Res. (2018) 40:564–72.

[27] Saitoh Y, Hirayama A, Kishima H, Shimokawa T, Oshino S, Hirata M, et al. . Reduction of intractable deafferentation pain due to spinal cord or peripheral lesion by high-frequency repetitive transcranial magnetic stimulation of the primary motor cortex. J Neurosurg. (2007) 107:555–9.

[28] Matsumura Y, Hirayama T, Yamamoto T. Comparison between pharmacologic evaluation and repetitive transcranial magnetic stimulation-induced analgesia in poststroke pain patients. Neuromodulation. (2013) 16:349–54.

[29] Kobayashi M, Fujimaki T, Mihara B, Ohira T. Repetitive transcranial magnetic stimulation once a week induces sustainable long-term relief of central poststroke pain. Neuromodulation. (2015) 18:249–54.

[30] Effect of high-frequency repetitive transcranial magnetic stimulation on chronic central pain after mild traumatic brain injury: A pilot study.Choi GS, Kwak SG, Lee HD, Chang MC J Rehabil Med. 2018 Feb 28; 50(3):246-252.

[31] Lin H, Li W, Ni J, Wang Y. Clinical study of repetitive transcranial magnetic stimulation of the motor cortex for thalamic pain. Medicine. (2018) 97:e11235.

[32] Jette F, Cote I, Meziane HB, Mercier C. Effect of single-session repetitive transcranial magnetic stimulation applied over the hand versus leg motor area on pain after spinal cord injury. Neurorehabil Neural Repair. (2013) 27:636–43.

[33] Yilmaz B, Kesikburun S, Yasar E, Tan AK. The effect of repetitive transcranial magnetic stimulation on refractory neuropathic pain in spinal cord injury. J Spinal Cord Med. (2014) 37:397–400.

[34] Quesada C, Pommier B, Fauchon C, Bradley C, Créac'h C, Vassal F, et al. . Robot-guided neuronavigated repetitive transcranial magnetic stimulation. (rTMS) in Central neuropathic pain. Arch Phys Med Rehabil. (2018) 99:2203–15.

[35] Misra UK, Kalita J, Tripathi GM, Bhoi SK. Is beta endorphin related to migraine headache and its relief? Cephalalgia. (2013) 33:316–22.

[36] Leung A, Shukla S, Fallah A, Song D, Lin L, Golshan S, et al. . Repetitive transcranial magnetic stimulation in managing mild traumatic brain injury-related headaches. Neuromodulation. (2016) 19:133–41.

[37] Shehata HS, Esmail EH, Abdelalim A, El-Jaafary S, Elmazny A, Sabbah A, et al. . Repetitive transcranial magnetic stimulation versus botulinum toxin injection in chronic migraine prophylaxis: a pilot randomized trial. J Pain Res. (2016) 9:771–7.

[38] Misra UK, Kalita J, Tripathi G, Bhoi SK. Role of beta endorphin in pain relief following high rate repetitive transcranial magnetic stimulation in migraine. Brain Stimul. (2017) 10:618–23.

[39] Fricova J, Klirova M, Masopust V, Novak T, Verebova K, Rokyta R. Repetitive transcranial magnetic stimulation in the treatment of chronic orofacial pain. Physiol Res. (2013) 62(Suppl 1):S125–34.

[40] Henssen DJHA, Hoefsloot W, Groenen PSM, Van Cappellen van Walsum AM, Kurt E, Kozicz T, et al. . Bilateral vs. unilateral repetitive transcranial magnetic stimulation to treat neuropathic orofacial pain: a pilot study. Brain Stimul. (2019) 12:803–5.

[41] Ahmed MA, Mohamed SA, Sayed D. Long-term antalgic effects of repetitive transcranial magnetic stimulation of motor cortex and serum beta-endorphin in patients with phantom pain. Neurol Res. (2011) 33:953–8.

[42] Malavera A, Silva FA, Fregni F, Carrillo S, Garcia RG. Repetitive transcranial magnetic stimulation for phantom limb pain in land mine victims: a double-blinded, randomized, sham-controlled trial. J Pain. (2016) 17:911–8.

[43] Ambriz-Tututi M, Alvarado-Reynoso B, Drucker-Colin R. Analgesic effect of repetitive transcranial magnetic stimulation. (rTMS) in patients with chronic low back pain. Bioelectromagnetics. (2016) 37:527–35.

[44] Cervigni M, Onesti E, Ceccanti M, Gori MC, Tartaglia G, Campagna G, et al. . Repetitive transcranial magnetic stimulation for chronic neuropathic pain in patients with bladder pain syndrome/interstitial cystitis. Neurourol Urodyn. (2018) 37:2678–87.

[45] Pinot-Monange A, Moisset X, Chauvet P, Gremeau AS, Comptour A, Canis M, et al. . Repetitive transcranial magnetic stimulation therapy. (rTMS) for endometriosis patients with refractory pelvic chronic pain: a pilot study. J Clin Med. (2019) 8:508.

[46] Pleger B, Janssen F, Schwenkreis P, Volker B, Maier C, Tegenthoff M. Repetitive transcranial magnetic stimulation of the motor cortex attenuates pain perception in complex regional pain syndrome type I. Neurosci Lett. (2004) 356:87–90.

[47] Picarelli H, Teixeira MJ, de Andrade DC, Myczkowski ML, Luvisotto TB, Yeng LT, et al. . Repetitive transcranial magnetic stimulation is efficacious as an add-on to pharmacological therapy in complex regional pain syndrome. (CRPS) type I. J Pain. (2010) 11:1203–10.

[48] Gaertner M, Kong JT, Scherrer KH, Foote A, Mackey S, Johnson KA. Advancing transcranial magnetic stimulation methods for complex regional pain syndrome: an open-label study of paired theta burst and high-frequency stimulation. Neuromodulation. (2018) 21:409–16.

[49] Ma SM, Ni JX, Li XY, Yang LQ, Guo YN, Tang YZ. High-frequency repetitive transcranial magnetic stimulation reduces pain in postherpetic neuralgia. Pain Med. (2015) 16:2162–70.

[50] Effects of high-frequency repetitive transcranial magnetic stimulation on reducing hemiplegic shoulder pain in patients with chronic stoke: a randomized controlled trial.Choi GS, Chang MC Int J Neurosci. 2018 Feb; 128(2):110-116.

[51] Irlbacher K, Kuhnert J, Ro¨richt S, Meyer BU, Brandt SA. Central and peripheral deafferent pain: Therapy with repetitive transcranial magnetic stimulation.Nervenarzt 2006;77(10):1196–203.

[52] Kalita J, Laskar S, Bhoi SK, Misra UK. Efficacy of single versus three sessions of high rate repetitive transcranial magnetic stimulation in chronic migraine and tension-type headache. J Neurol. 2016;263:2238–2246.

[53] Pei Q, Wu B, Tang Y, Yang X, Song L, Wang N, et al.Repetitive transcranial magnetic stimulation at different frequencies for postherpetic neuralgia: a double-blind, sham-controlled,randomized trial. Pain Physician 2019;22:E303–13.

[54] Andre-Obadia, N, Magnin, M, Garcia-Larrea, L. On the importance of placebo timing in rTMS studies for pain relief. Pain 2011;152:1233–7.

[55] Lefaucheur, JP, Drouot, X, Nguyen, JP. Interventional neurophysiology for pain control: duration of pain relief following repetitive transcranial magnetic stimulation of the motor cortex. Neurophysiol Clin 2001;31:247–52.

[56] Teo WP, Kannan A, Loh PK, Chew E, Sharma VK, Chan YC. Tolerance of motor Cortex rTMS in chronic migraine. J Clin Diagn Res. 2014;8:MM01-02

[57] Theta burst stimulation in the treatment of chronic orofacial pain: a randomized controlled trial.Kohútová B, Fricová J, Klírová M, Novák T, Rokyta R Physiol Res. 2017 Dec 20; 66(6):1041-1047.

[58] Melchior C, Gourcerol G, Chastan N, et al. Effect of transcranial magnetic stimulation on rectal sensitivity in irritable bowel syndrome: a randomized, placebo-controlled pilot study. Colorectal Dis. 2014 Mar;16(3):O104-11.

[59] Hosomi K, Kishima H, Oshino S, Hirata M, Tani N, Maruo T, Yorifuji S, Yoshimine T, Saitoh Y. Cortical excitability changes after high-frequency repetitive transcranial magnetic stimulation for central poststroke pain. Pain 2013; 154:1352-1357

[60] Khedr EM, Kotb H, Kamel NF, Ahmed MA, Sadek R, Rothwell JC. Long-lasting analgic effects of daily sessions of repetitive transcranial magnetic stimulation in central and peripheral neuropathic pain. J Neurol Neurosur Ps 2005; 76:833-838.

[61] Effects of low versus high frequencies of repetitive transcranial magnetic stimulation on cognitive function and cortical excitability in Alzheimer’s dementia. Ahmed MA, Darwish ES, Khedr EM, El Serogy YM, Ali AM. J. Neurol. 2012;259:83–92.

[62] Andre-Obadia N, Mertens P, Lelekov-Boissard T, et al. Is Life better after motor cortex stimulation for pain control?Results at long-term and their prediction by preoperative rTMS. Pain Physician. 2014;17:53–62.

[63] Johnson S, Summers J, Pridmore S. Changes to somatosensory detection and pain thresholds following high frequency repetitive TMS of the motor cortex in individuals suffering from chronic pain.Pain 2006;123:187-92.

[64] Malavera M, Silva F, Garcia R, Quiros J, Dallos M, Pinzon A. Effects of transcranial magnetic stimulation in the treatment of phantom limb pain in landmine victims: a randomized clinical trial. Journal of the Neurological Sciences 2013;333:e534.

[65] Lang M, Treister R, Klein MM, et al. Repetitive transcranial magnetic stimulation (rTMS) of the primary motor cortex for treating facial neuropathic pain-preliminary results of arandomized, sham-controlled, cross-over study. Mol Pain.2014;10(Suppl 1):P6.

[66] Lefaucheur JP, Menard-Lefaucheur I, Goujon C, et al.Predictive value of rTMS in the identification of responders to epidural motor cortex stimulation therapy for pain. J Pain. 2011;12:1102–1111.

[67] Kalita J, Bhoi SK, Misra UK. Effect of high rate rTMS on somatosensory evoked potential in migraine. Cephalalgia. 2017;37:1222-1230.

[68] Lefaucheur et al. 2003 The authors of the systematic review did not provide references

[69] Melchior et al. 2013 The authors of the systematic review did not provide references

[71] Goudra 2017 The authors of the systematic review did not provide references

Khedr EM, Kotb HI, Mostafa MG, Mohamad MF, Amr SA, Ahmed MA, Karim AA, Kamal SM. Repetitive transcranial magnetic stimulation in neuropathic pain secondary to malignancy: a randomized clinical trial. Eur J Pain. 2015 Apr;19(4):519-27. doi: 10.1002/ejp.576. Epub 2014 Aug 21. PMID: 25142867.

[72] Saitoh Y, Hirayama A, Kishima H, Oshino S, Hirata M, Kato A, Yoshimine T. Stimulation of primary motor cortex for intractable deafferentation pain. Acta Neurochir Suppl. 2006;99:57-9. doi: 10.1007/978-3-211-35205-2_11. PMID: 17370765.
